# Surface-supported metal−organic frameworks with geometric topological diversity via scanning tunneling microscopy

**DOI:** 10.1016/j.isci.2024.109392

**Published:** 2024-03-04

**Authors:** Xiaoyang Zhao, Xinrui Miao

**Affiliations:** 1 College of Materials Science and Engineering, South China University of Technology, Guangzhou 510640, People’s Republic of China

**Keywords:** Materials characterization, Materials chemistry

## Abstract

Surface-supported metal−organic frameworks (SMOFs) are long-range ordered periodic 2D lattice layers formed by inorganic metal nodes and organic ligands via coordination bonds on substrate surfaces. The atomic resolution STM lays a solid foundation for the conception and construction of SMOFs with large area, stable structure, and special function. In this review, the cutting-edge research of SMOFs from design strategy, preparation process, and how to accurately achieve structural and functional diversity are reviewed. Furthermore, we focus on the design and construction of novel and fascinating periodic and fractal structures, in which some typical honeycomb structures, Kagome lattice, hexagonal geometry, and Sierpiński triangles are summarized, and the related prospects for designing functional nanoscale systems and architectures are prospected. Finally, the challenges faced in the design and synthesis of SMOFs are denoted, and the application prospect and development trend of SMOFs are forecasted based on the current research status.

## Graphical abstract

**Figure:**
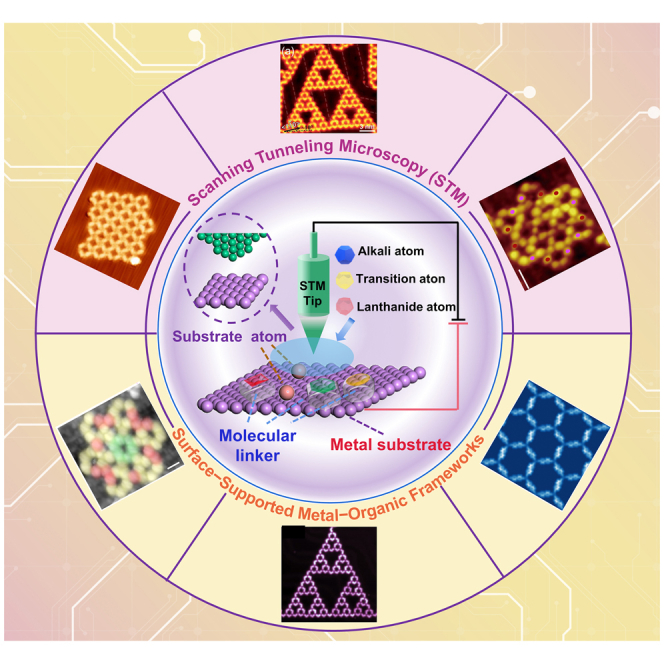

## Introduction

Metal−organic frameworks (MOFs) are a class of porous materials that are composed of metal ions or clusters and organic ligands connected by coordination bonds.^1^^,^^2^^,^^3^ In recent years, MOFs have attracted more and more attention because of their novel structures and their extensive applications in gas adsorption, luminescence, chemical sensing, magnetism, and heterogeneous catalysis.^4^^,^^5^^,^^6^^,^^7^ This field has significant implications for the design and fabrication of novel functional materials, such as porous networks, molecular switches, sensors, and catalysts.^8^^,^^9^^,^^10^^,^^11^

On-surface chemistry has enabled the formation of two-dimensional (2D) MOFs with atomic thickness on an appropriate surface, which are also called surface-supported metal−organic frameworks (SMOFs) materials.^12^ The embedding and rationalization construction of metal centers in tailored surface environments is an important way to construct SMOFs with unique structure and excellent physical and chemical properties. In general, metal centers are derived from the deposition of various extrinsic metal atom, such as transition metals, alkali metals, and lanthanide metals, which are deposited on the surface to form SMOFs with organic ligands.^13^^,^^14^ Based on this method, SMOFs with various sizes and symmetries are broadly synthesized. However, on-surface reactions also show that the addition of external metal atoms is not necessary for the synthesis of SMOFs. The metal atoms in SMOFs can be derived from the adatoms of the substrates.^15^^,^^16^ Ullmann coupling is a typical example, where metal surface-induced dehalogenation of precursors can trigger the spontaneous construction of organometallic frameworks, and connect via C−M−C bonds, where M are surface metal atoms.^17^^,^^18^^,^^19^ Furthermore, apart from the Ullmann reaction, SMOFs can also be obtained after dehydrogenation of carboxyl, hydroxyl and sulfate groups after annealing at a certain temperature.^20^^,^^21^^,^^22^ Therefore, metal atoms have far-reaching significance for the regulation of the structure and function of 2D networks on the nanometer scale.

The precise controllable structure of SMOFs is an important way to realize specific functions and applications. In recent years, SMOFs with diverse periodic and fractal structures have been successfully prepared, including honeycomb structure, Kagome lattice, hexagonal geometry and Sierpiński triangles, etc., which greatly enrich the diversity of SMOFs functions and topologies.^23^^,^^24^^,^^25^^,^^26^^,^^27^

Scanning tunneling microscopy (STM) is a technique that can directly observe the surface of materials at the atomic scale, by measuring the tunneling current between a sharp tip and the sample.^28^^,^^29^^,^^30^^,^^31^ STM has great importance for the study of nanoscience and nanomaterials, as it can reveal the surface morphology, the local density of states, and the manipulation of atoms. STM also enables the structural analysis and imaging formation of biological molecules.^32^^,^^33^^,^^34^ STM lays a solid foundation for the design and construction of SMOFs with large area, stable structure and special function.^35^^,^^36^^,^^37^^,^^38^^,^^39^^,^^40^^,^^41^

**Figure 1:**
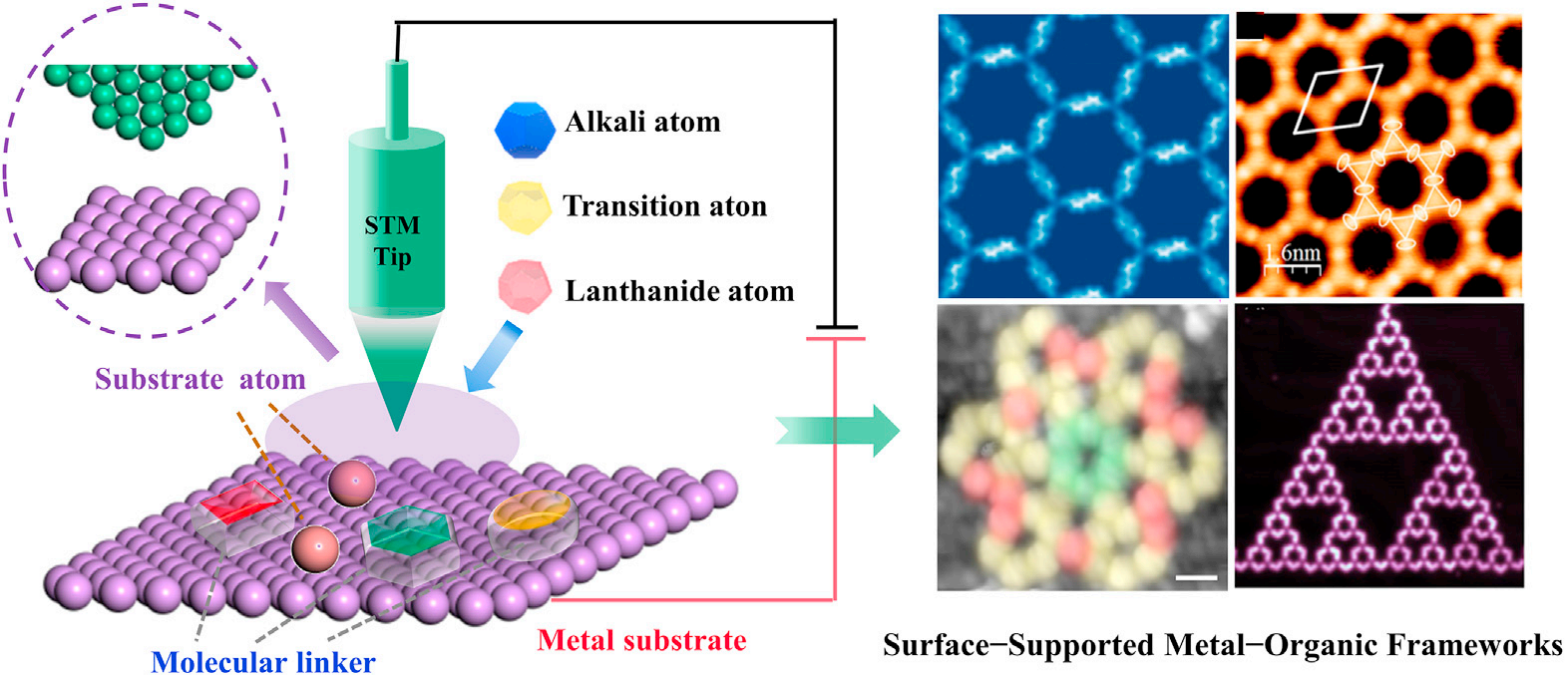
Two construction strategies and several typical topologies of SMOFs SMOFs constructed by extrinsic atom induced and substrate-assisted assembly; the fascinating periodic and fractal structures including honeycomb structure, Kagome lattice, hexagonal geometry, and Sierpiński triangles.

In this review, The SMOFs constructed by extrinsic atom induced and substrate-assisted assembly are reviewed. On this basis, the complex and fascinating periodic and fractal structures formed by on-surface self-assembly are summarized, including honeycomb structure, Kagome lattice, hexagonal geometry and Sierpiński triangles. The prospect of designing diversified nanosystems and structures is proposed (Figure 1). In this process, the discussions are mainly based on the experimental and analytical results of STM, and are often supplemented by theoretical calculation and simulation analysis using density functional theory (DFT). Finally, we denoted the challenges faced in the design and synthesis of SMOFs, and the application prospect and development trend of SMOFs are forecasted based on the current research status.

## Extrinsic metal atoms induced SMOFs construction

### Transition metal

Nanotechnology is ongoing pursuit to manipulate the magnetic properties of single and multiple atoms. The magnetic property of a transition−metal atoms are strongly influenced by the electron configuration of the orbitals around them.^42^ Nanoscale pores formed by transition metal atoms and organic molecules can serve as selective adsorption sites for specific atoms or molecules. If there is electron coupling between metal atoms and organic molecules, the introduction of metal atoms will regulate the molecular orbitals of organic molecules. When the electronic coupling between metal atoms and organic molecules is strong enough, and the conjugation of organic molecules is relatively favorable, novel band structures predicted by theory will be generated, such as organic topological insulators. Single transition metal atoms and organic ligands are usually coordinated in a 2-fold, 3-fold, 4-fold, or 5-fold geometry on the surface. The metal atoms in these 2D coordination structures are not fully coordinated and their top action node are available for further binding.^43^^,^^44^^,^^45^ This mononuclear transition atom configuration is favored for magnetism, catalysis, and gas adsorption. Liljeroth et al.^46^ deposited mononuclear metal atoms (iron or nickel) and 9,10-dicyananthracene on ultrathin insulating surfaces to form linear structures. The frontier molecular orbitals were directly visualized by STM orbital imaging. Combined with DFT calculations, the distribution of electrons around the complex was inferred. The experiment revealed how the sequence of the electronic orbits and the spin−state of the MOF could be transformed by reasonable customization of metal center. High-spin Fe-MOF had a single−occupancy delocalized orbit with big spin-splitting and was promising as a module for molecular spintronics.

Complexes with dinuclear coordination centers are widely studied in biomedicine and traditional 3D coordination chemistry. Such as dinuclear metal centers in various metalloproteins, this special configuration makes a critical difference in respiration or metabolism, thus attracting wide interests in biomimetic catalysts.^47^^,^^48^^,^^49^ In SMOFs systems, however, dinuclear coordination centers are relatively rare. A famous example is the assembly of iron and cobalt as bimetallic centers binding ligand molecules on the substrate surface.^50^ It was found that the di-iron center was more active for the O_2_ dissociation effect than the mono-iron, which was attributed to the higher reactivity of the exposed metal atoms. In addition, they provided sites for axial connections, which could extend the 2D network into 3D structures.

**Figure 2:**
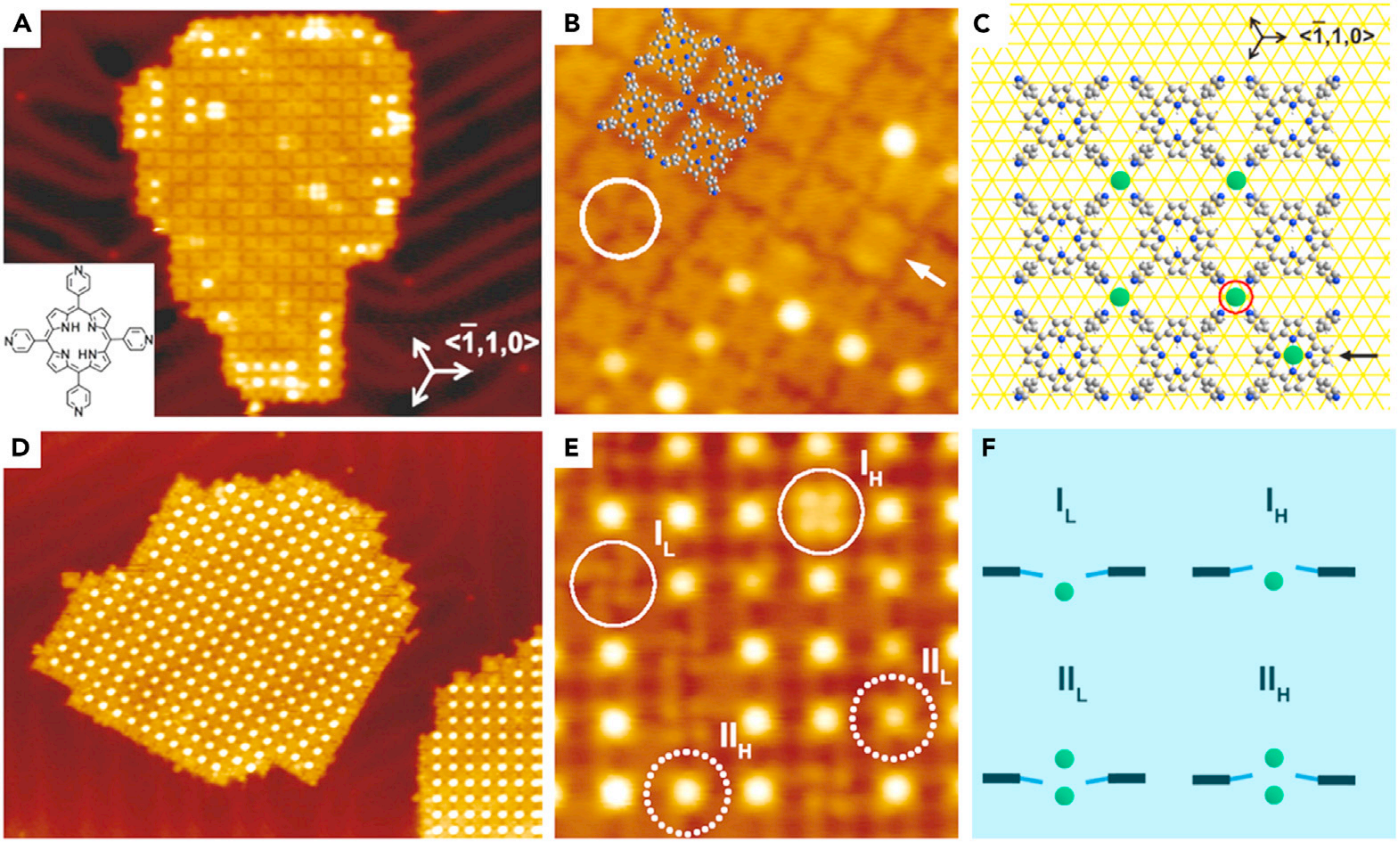
Forming an extended 2D network by TPyP and the binuclear metal Fe (A) STM pattern of a 2D network formed by ligand molecule Fe atoms. (B) STM image of the clover structure. (C) Calculation model of 2D network structure. (D) Network structure under high concentration of Fe source. (E) High-resolution STM image with different connection types. (F) Theoretical model of four types of intersection points. Figures reproduced from: Lin et al.^51^ Copyright, 2014, American Chemical Society.

After that, the self-assembly of 5,10,15,20-tetra-(4-pyridyl)porphyrin (TPyP) and Fe on Au(111) substrate was reported.^51^ The experimental results showed that TPyP and binuclear metal Fe form an extended 2D network. After adding small amounts of Fe and then heating them up to 423 K, networks with almost square lattice appeared as presented in Figure 2A. An interesting clover shape was carefully observed at a particular junction (Figures 2B and 2C). The high-resolution STM image showed four types of connections (Figures 2D and 2E). Several unique and novel assembly patterns were presented in detail in Figure 2F. Interestingly, the network island composed of hundreds of molecules and atoms could move laterally on Au(111) substrate without destruction by STM tip, which was due to the strong coordination interaction between Fe atoms and ligands. Furthermore, the low-temperature STM revealed the Kondo resonance around the Fe atom. The out-of-plane binuclear coordination center provided diversified functions, such as catalysis, magnetic and template of framework architectures. In the Ni-TPyP network, the binuclear Ni center was located on the Au(111) surface, where the top-Ni atom was located above the molecular plane. The attachment of H atom not only increased the spin state and zero-field splitting energy of NiH_x_ species, but also changed the direction of magnetic anisotropy between NiH and NiH_2_. This work provided new insights into the structure-activity relationship and rational regulation at the micro level.^52^

Polynuclear complexes−where organic ligands connect to more than one metal atom are significant in practical applications because of their orbital electron arrangement or magnetic properties and potential for functional reactivity pathways. In SMOFs, multiple active metal centers can enhance the catalytic efficiency compared with mononuclear metals. On the other hand, the magnetic and electrical interactions between metal atoms can produce many novel properties, such as synergistic electron and steric hindrance effect.^53^^,^^54^ However, it is still challenging to synthesize multicore SMOFs on the surface. Krull et al.^55^ reported 1D metal−organic nanostructures synthesized by terpyridine (tpy)-based molecules and tri-iron node. SMOFs consisting of plane-structured tpyʼs were connected via a three Fe^3+^ clusters. Interestingly, this coordination interaction was accompanied by the redistribution and arrangement of charges and electrons, which not only stabilized the coordination bonds, but also possibly generated catalytic activity located in multinuclear centers. The results positioned on surface supramolecular chemistry as a route to synthesize functional multinuclear coordination nodes, opening the door for nanomaterials with new functions.

The introduction of polynuclear transition metal has been proved to be an effective strategy for multi-functional *in situ* regulation of SMOF structures. Hence, dimension-related structural transformations especially from higher to lower dimensions are investigated. In addition, the corresponding mechanisms are of great significance for artificially fabricating nanostructures with controllable dimensions on surfaces. For example, 1-D chains introduced by Ni atoms could be transformed into 0-D triangular clusters in the MOF chain system formed by the deposition of cytosine molecules on the Au(111) surface at RT.^56^ Three stable cytosine molecules were coordinated with three Ni atoms to form the clusters discretely distributed on the surface. Due to the uneven distribution of binding sites on cytosine molecules, the coordination of cytosine molecules and Ni atoms completely screened the available binding sites within the triangular clusters, inhibiting the formation of any potential coordination bonds or hydrogen bonds between clusters. The intermolecular repulsion force between clusters finally led to the discrete distribution of clusters. This structural transition from 1-D to 0-D was the result of the introduction of polynuclear transition metals.

### Alkali metal

Most of the current SMOFs are established on *d*-block transition metal atoms. However, the flexibility of these SMOFs has not been well demonstrated. One important reason is that *d*-block atoms tend to form highly symmetrical stable structures when assembled on the surface based on the fixed direction of the peripheral electron orbitals.^57^^,^^58^ In contrast, alkali metal ions are completely isotropic in ionic bonding, which facilitates coordination with organic molecules to construct flexible and tunable SMOFs. The construction of flexible SMOFs via alkali metal ions usually demands accurate regulation of the bonding configuration between the nucleophilic group of the ligand and the metal ion, resulting the coordination number of coordination sites is the same, while maintaining considerable flexibility.^59^^,^^60^

**Figure 3:**
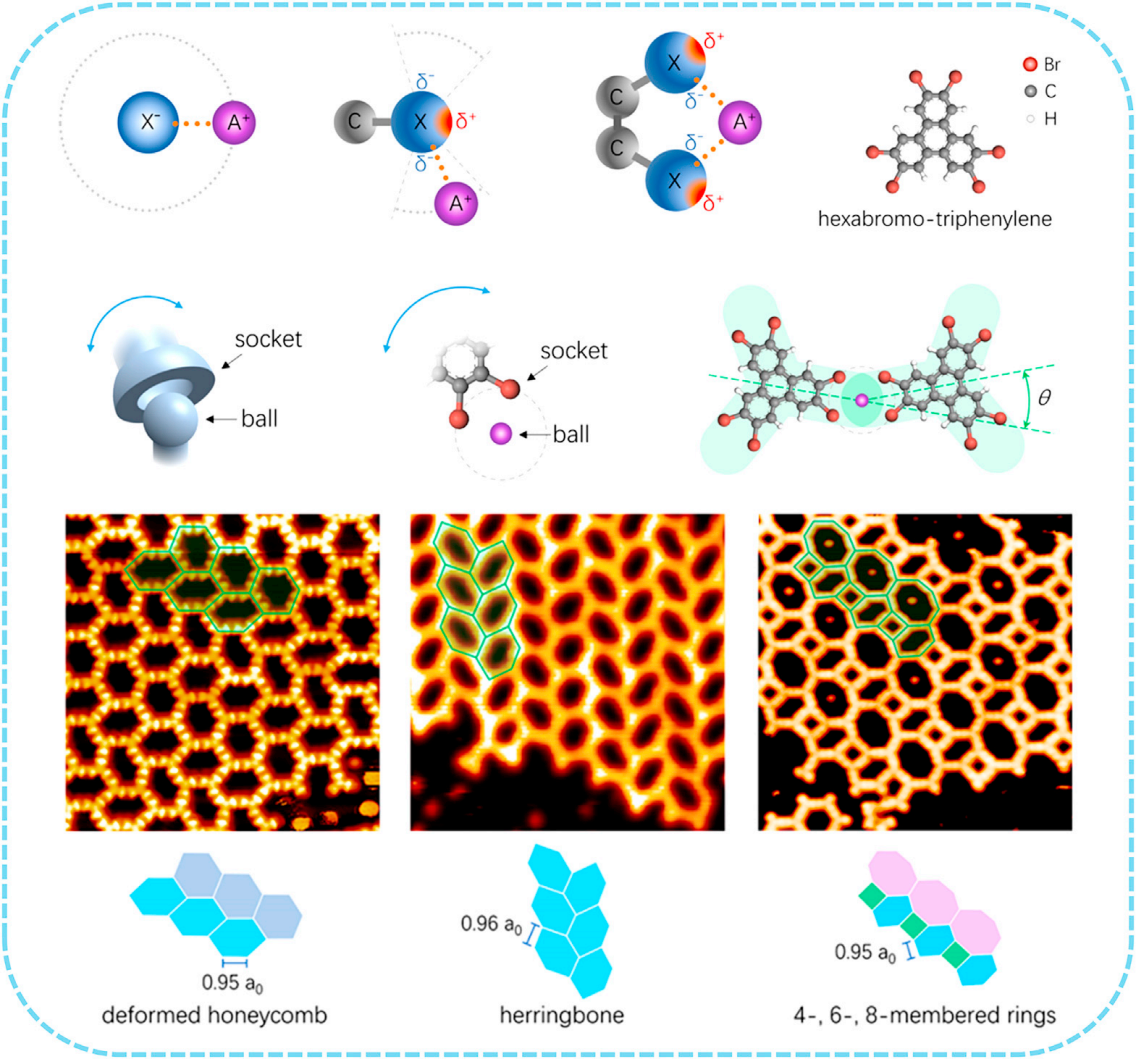
Construction of Na-based SMOF A design strategy of SMOF based on alkali metal and halogen−containing molecule and its construction on Au(111) substrate. Figures reproduced from: Shan et al.^61^ Copyright 2021 American Chemical Society.

Flexible alkali−halogen bonds can be utilized to design and construct adjustable SMOFs by different alkali atoms and halogen−containing molecules. In 2021, A Na-based SMOF was successfully constructed by a 3-fold ring ligand on Au(111) surface (Figure 3).^61^ Low−temperature STM revealed its flexible and diverse lattice. In these 2D structures, the deflection angle of the metal atom junction site changed over a large range, which was rare reported in previous studies. Alkali metal ions and halogen ions formed strong ionic bonds by electrostatic attraction of electric charge, and exhibited isotropy and non-directivity. Halogen atoms, on the other hand, had high electron density and electronegative electron donor sites, which were liable to coordinate with metal ions. This work pointed out a feasible route toward building and synthesizing more tunable functional SMOFs.

**Figure 4:**
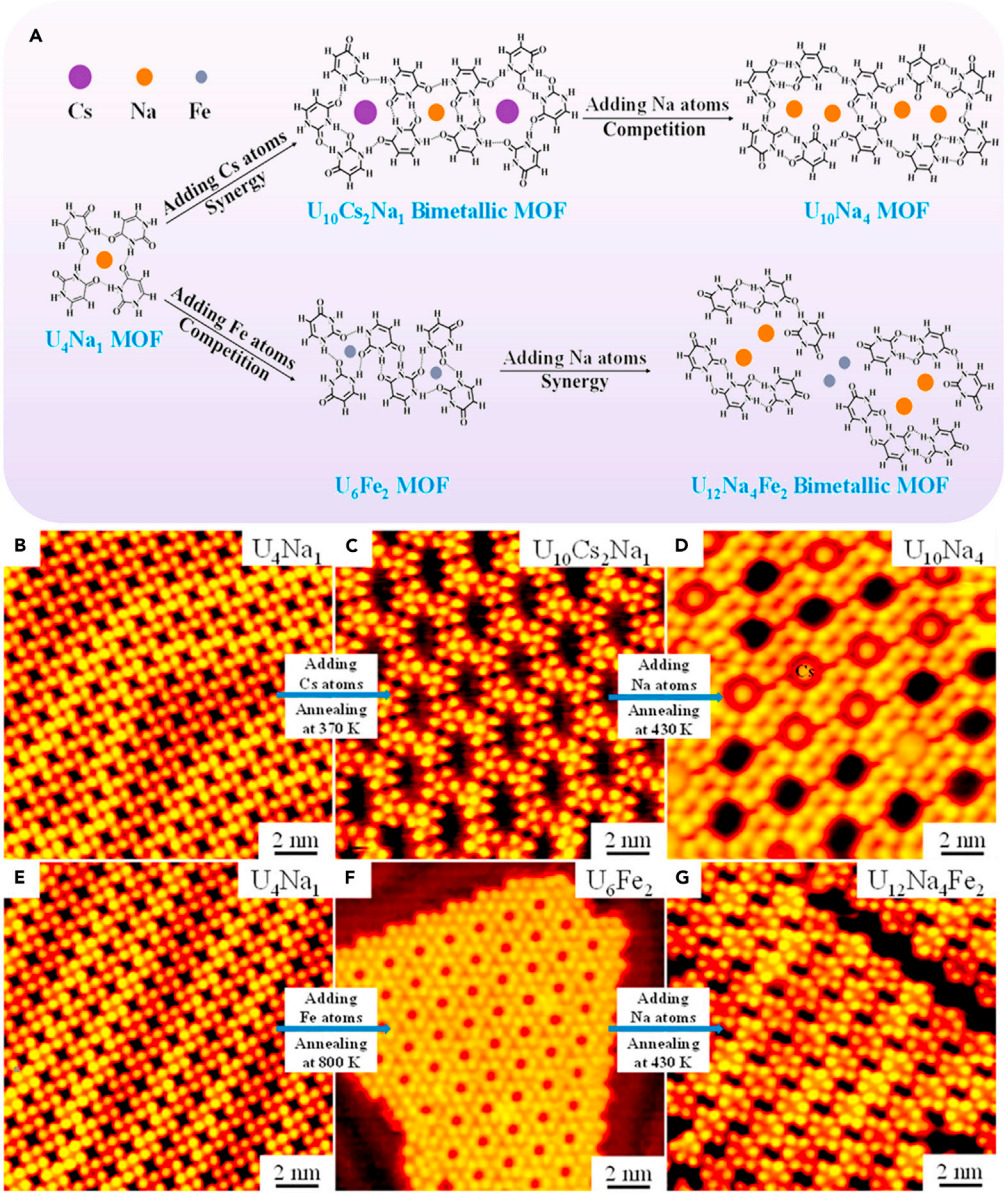
Construction of bimetallic SMOF (A) Formation of different SMOFs involving mixed metal components. (B−E) Large-scale STM images displaying the structural transformations from U_4_Na_1_ via U_10_Cs_2_Na_1_ to U_10_Na_4_ SMOFs. (E−G) Large-scale STM images displaying the structural transformations from U_4_Na_1_ via U_6_Fe_2_ to U_12_Na_4_Fe_2_ SMOFs. Figures reproduced from: Li et al.^62^ Copyright 2021 American Chemical Society.

Here, it needs to be emphasized that building a system involving mixed metal components have proved to be a potential strategy in recent years, which will promote our understanding of manufacturing bimetallic SMOF and explore unprecedented features and properties. The RNA base uracil (U) molecule and alkali metal Na were deposited on the Au(111) substrate to form a single metal SMOF (U_4_Na_1_) (Figure 4A).^62^ The introduction of Cs atoms into the system could produce a bimetallic SMOF composed of U_10_Cs_2_Na_1_ motifs through the cooperation and competition between ionic bonds and intermolecular hydrogen bonds (Figures 4B−4D). Furthermore, the transition metal Fe was introduced into the U_4_Na_1_ structure, and a porous network structure (U_6_Fe_2_) was formed after annealing at 800 K. More interestingly, another mixed metal network structure (U_12_Na_4_Fe_2_) was obtained by further depositing Na atoms on the U_6_Fe_2_ pre-covered surface and then annealing at 430 K (Figures 4E−G). These facts indicated that the alkali metal could be replaced by another alkali metal or transition metal in the construction of SMOFs. The systematic study of this kind of metal was helpful to explore the nature of the interaction mechanism of non-covalent bond. The preparation strategy of bimetallic SMOF will pave the way for further study of the structural diversity of SMOF and the realization of unprecedented functions and properties.

### Lanthanide metal

Lanthanide, also known as rare earth, is a kind of multi-functional inorganic metal element that is widely used in a variety of high-tech fields, including polishing materials, light−emitting diodes, magnetic materials, luminescence sensors, and biological imaging, due to their unique orbital energy levels and outer electron arrays. To date, most SMOFs depended on transition metal atoms as the center. In recent years, lanthanide−directed assembly of SMOFs has gradually entered the field of vision, and it has development potential in guiding the formation of complex metallosupramolecular nanosystems on surfaces.

The functional characteristics of lanthanide elements are determined by the special arrangement of *f*-electrons, big atomic radius and intrinsic large spin orbit coupling. Their assembly and reasonable design in the interface environment are key factors to establish the relationship between the structures and physicochemical properties, resulting in new functions of technical significance.

Lanthanide metal ions, as hard Lewis acids, have powerful binding force for nitrile, pyridine and carboxylic acid donors. The 4-fold and 5-fold pattern are established by europium (Eu) atoms and 2,2’:6′,2″-terpyridine and carbonitrile molecules on an Au(111) substrate. Under the reasonable control of ligand concentration, 1D and 2D self-assembled structures are fabricated. Urgel et al.^63^ reported that nano-porous grids could assemble on Cu(111) surface using carboxylic acid ligands and Gd atoms observed by means of STM under different conditions. The experiment result confirmed the coordination bonds are not dominated by covalent components, which was also reflected in remarkable stability of SMOF structure. This research introduced an emerging metal supramolecular system with the function of *f*-block substances.

**Figure 5:**
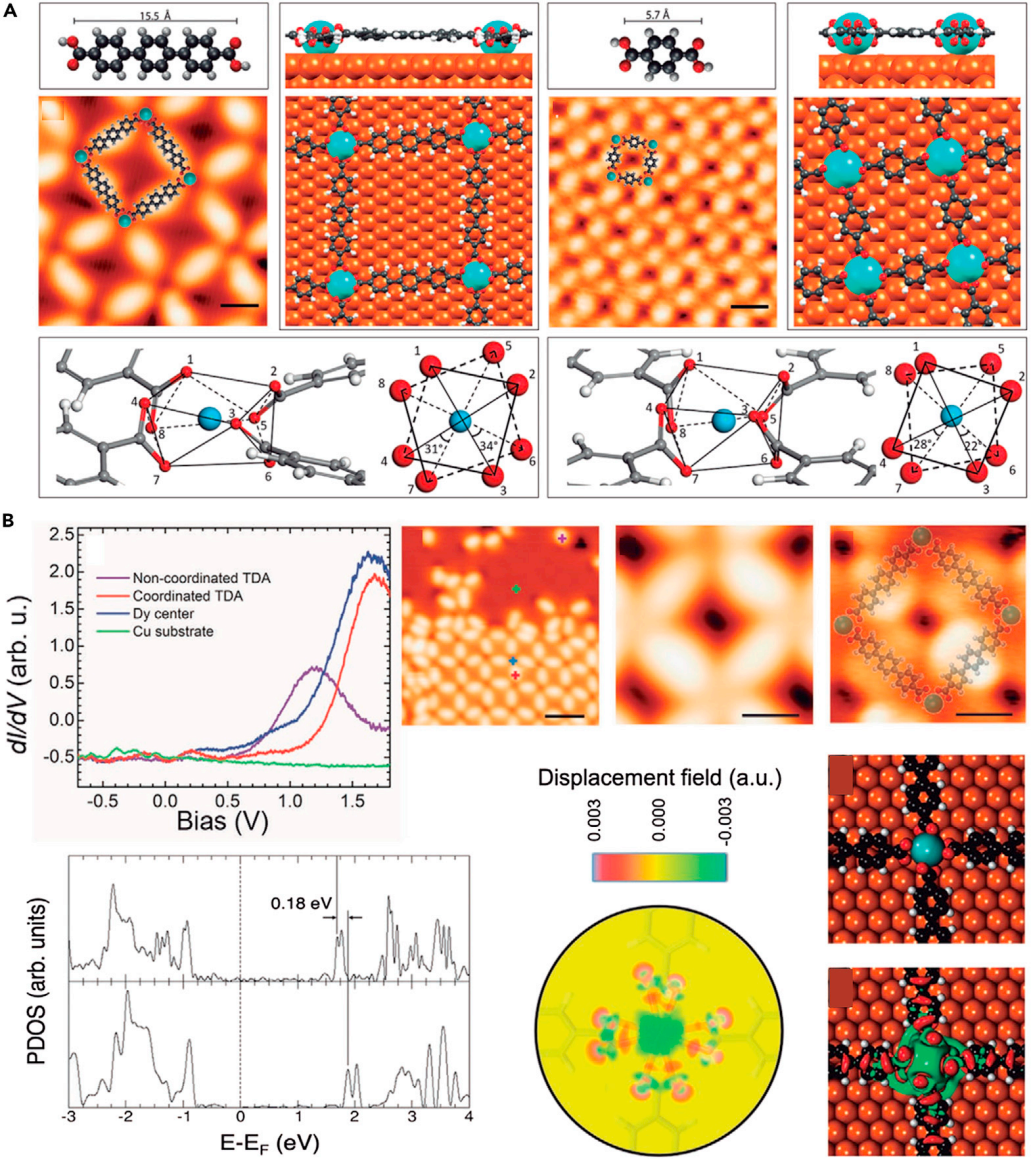
Design and synthesis of lanthanide SMOF (A) SMOF constructed on Cu(111) surface based on lanthanide Dy and carboxylic acid ligands. (B) Electronic structure of Dy-based SMOF on Cu(111). Figures reproduced from: Parreiras et al.^64^ Copyright 2021 Wiley.

In 2021, the electronic and magnetic properties of SMOFs constructed by lanthanide element Dy and carboxylic acid ligands on Cu(111) were investigated (Figure 5A).^64^ The surface-supported metal−organic coordination could cause a transform in the direction of the effortless magnetization axis of the metal coordination center, and significantly increased the magnetic anisotropy. Theoretical calculations revealed that the direction was the result of a precise adjustment between the bonding mode between the Dy and O atoms and the accurate geometry of the crystal field (Figure 5B). This opened up a distinctive way to regulate the magnetic anisotropy and magnetic torque of surface rare earth elements.

In addition to pyridine and carboxylic acid, tetrapyrrole ligands have unique advantages in the construction of SMOFs. Metal tetrapyrrole is widely used in sensing, photovoltaic, catalysis and quantum technology. Although the formation of 3D metal tetrapyrrole complexes has been deeply studied, the exploration of *in-situ* incorporation of lanthanide metals at 2D interfaces are still in their infancy. The establishment of surface tetrapyrrole metallization protocol in ultra-high vacuum (UHV) environment is a general method for metal−organic complexes and nanostructure engineering. Recently, Felix et al.^65^ have studied the intermediate complexes formed during the metallization of tetrapyrrole with lanthanide atoms, supplemented by DFT calculations. Free base 5,10,15,20-tetraphenylporphyrin (2H-TPP) and lanthanide metal Ce were deposited on Ag(111) sequentially and induced by thermal annealing or STM tip operation. STM could obviously observe the metallization process of TPP array, reflecting a multi-step reaction pathway. This work contributed to the understanding the interaction between lanthanide metals and tetrapyrrole, and provided a profound insight for the formation and functional application of lanthanide metal SMOFs on the surface.

Most lanthanide-directed SMOFs are limited to mononuclear metals, and the research on the assembly structure, electronics and magnetism of multi-nuclear lanthanide oriented SMOFs is still in its infancy. The design of multi-nuclear lanthanide network is an emerging field, which shows great potential in the fields of nanomagnetism, spintronics and quantum information. Therefore, it is crucial to design multi-nuclear lanthanide SMOFs and explore its structure and properties.

A tunable internodal distance network structure could be constructed on Cu(111) surface by using carboxyl-terminated organic connectors *p*-terphenyl-4,4″- dicarboxylic acid (TDA) and binuclear Dy clusters.^66^ The thermal annealing treatment of the reticulated Dy-TDA structure formed an unprecedented hexagonal grid structure on account of binuclear Dy clusters, showing a particular 6-fold Dy···O−bond motif. All metal supramolecular structures were stable at room temperature. This work provided profound insights into the surface self-assembly of binuclear lanthanide metals. In 2022, Moreno et al.^67^ synthesized lanthanide-directed binuclear metals (Er and Dy) SMOFs on coinage metals, which were inspected and rationalized via STM, STS, DFT and multiplet calculations. Er and Dy exhibited the same +3 oxidation state, but the energy level alignment of their unoccupied molecular orbitals had changed. When the metal center was replaced from Er to Dy, the easy axis of magnetization was reoriented and the magnetic anisotropy was also increased.

**Figure 6:**
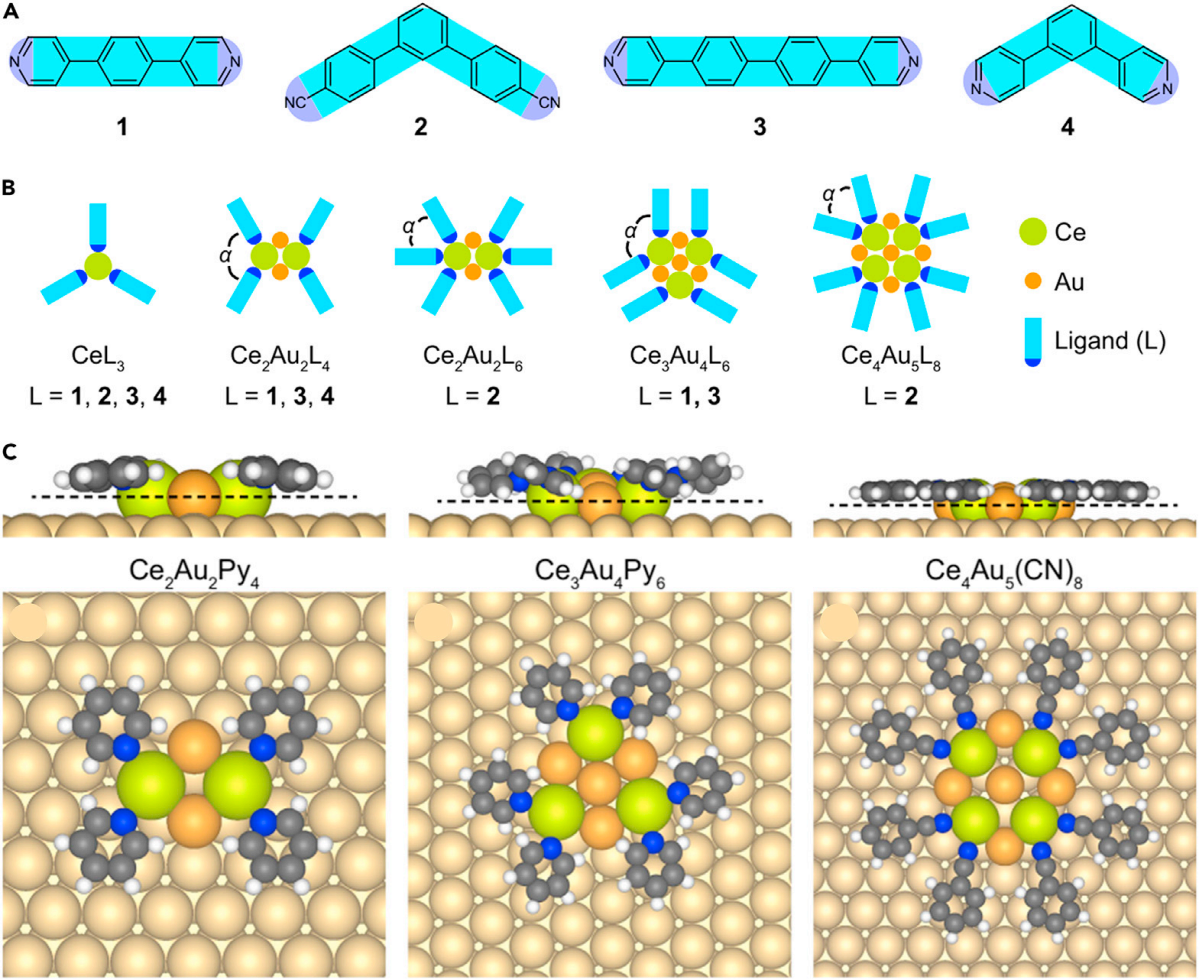
Forming SMOF by bridging Ce clusters with Au atoms (A) Chemical structures of the pyridine and nitrile ligands. (B) Theoretical model of the coordination motifs. (C) Theoretical models of different forms of multinuclear coordination structures. Figures reproduced from: Liu et al.^68^ Copyright 2021 Nature Publishing Group.

Liu et al.^68^ realized the regular coordination assembly of Ce clusters bridged by Au atoms on surface using different pyridine and nitrile ligands (Figure 6A). Figure 6B showed a schematic diagram of intermolecular bonding. The DFT calculation revealed that the interaction between lanthanide Ce and substrate. Au played a crucial role in the construction of Ce metal clusters. (Figure 6C). STM showed that the Ce metal atoms close to each other were connected by Au atoms. On this basis, Ce and the organic ligand assembled into regular and ordered nanostructures based on the coordination, resulting in the destruction of Ce−Au interaction.

In recent years, the assembly strategy of orthogonal coordination interactions to solve the design of metal supramolecular structures have been extensively studied. Combined with the synergistic action of lanthanide and transition elements centers in a specific environment, the unique structure and properties are achieved by the combination of *3d*ays and *4f* elements and the embedding of nanoscale. Such systems have promising applications in heterogeneous catalysis, molecular spintronics or luminescent devices and engineering magnetic response.

A *d-f* bimetallic networks was successfully engineered on a well-defined Ag (111) surfaces based on an orthogonal combination of two metal centers.^69^ The *in-situ* observation process of the heterostructure was explored by STM. The porphyrin modules self-assembled into a tetragonal close-packed structure after deposited on Ag(111) surface, and then a porous structure stabilized by 4-fold Gd···NC interaction was formed when Gd atoms were co-deposited. The co-deposition of Co atoms led to the selective metallization of central tetrapyrrole macrorings, forming a unique *d-f* array. This study opened new avenues to the design of surface metal supramolecules, thereby advancing the development of heterometallic SMOF.

## Substrate−assisted SMOFs construction

SMOFs are usually synthesized by the co-adsorption of organic ligands (such as pyridine, carboxylic acid derivatives) and metal atoms on the surface. SMOFs with disparate symmetries and crystal structures are established based on the strategy. Further studies on-surface reactions demonstrate that deposition of extrinsic metals is not the only way to construct SMOFs. The metal source in SMOFs can also be replaced by the atoms of the substrate.

Substrate−assisted SMOFs typically exist in two forms, one represents the final product constructed through on-surface reactions or molecular assembly.^70^^,^^71^ For instance, a stable SMOF with Kagome network structure was formed using porphyrin (TPyP) as building blocks on the Au(111) surface.^72^ The other is organometallic intermediate which is synthesized through on-surface reaction by Ullmann coupling. The intermediate products of Ullmann reactions on metal surfaces are often constituted by the interaction of dehalogenated carbon radicals with metal atoms.^73^^,^^74^ Thus, SMOFs can be constructed by annealing halogen-containing precursors. Wang et al. combined with STM and DFT calculation, provided direct evidence of organometallic intermediates, and the free radical dimer connected by C−Cu−C bridge was formed.^75^

It is worth noting that even for the same reactant, the assembly structure can be greatly different on different metal substrates. The assembly process of 3,6-dibromo-9,10-phenanthrene quinone (DBPQ) molecules on two different metal substrates was systematically investigated.^76^ STM showed that DBPQ were assembled into chiral molecular rows on the Ag(100) surface by multiple hydrogen and halogen bonds at room temperature. After thermal annealing to 573 K, DBPQ on the Ag(100) surface formed SMOFs, which was composed of metal−organic intermediates of Ulmann-like reaction. In contrast, the more active Ag(110) surface catalyzed the dissociation of C−Br bonds in DBPQ, but also limited surface dispersion, leading to the formation of tanglesome network structure on the substrate.

On the surface, the C−M−C linkages assume mostly straight alignments, and although the reversibility of C−M−C bonds promotes structural balance, highly curved organometallic bonds tend to produce topological defects. It is an effective way to reduce the flexibility of C−M−C bond angle by introducing steric hindrance. For instance, the introduction of a large amount of o-methyl to improve the steric hindrance of Cu(*p*-bromophenyl) benzene precursor could reduce the structural defects of SMOFs on Cu(111) and Ag(111). DFT calculation showed that the curved C−Cu−C bond was more favorable in energy, which facilitates the formation of stable SMOFs.^77^

In addition to dehalogenation, SMOFs can be constructed by dehydrogenation of carboxyl group, hydroxyl group, amino group and sulfhydryl group. Chi et al.^78^ employed the triple symmetric 1,3,5-tris(4-aminophenyl)benzene (TAPB) as a precursor to construct SMOFs on Cu(111) surface by the dehydrogenation of aromatic amines. STM revealed that the 2D porous network was formed by the N−Cu−N interaction between dehydroamino and copper atoms. In addition, SMOF was a triple symmetric structure composed of three kinds of hexagons, which was closely related to the precise places of the separated hydrogens of the −NH_2_. The results showed that the accurate regulation of the heating process was a powerful means to establish specific structures.

**Figure 7:**
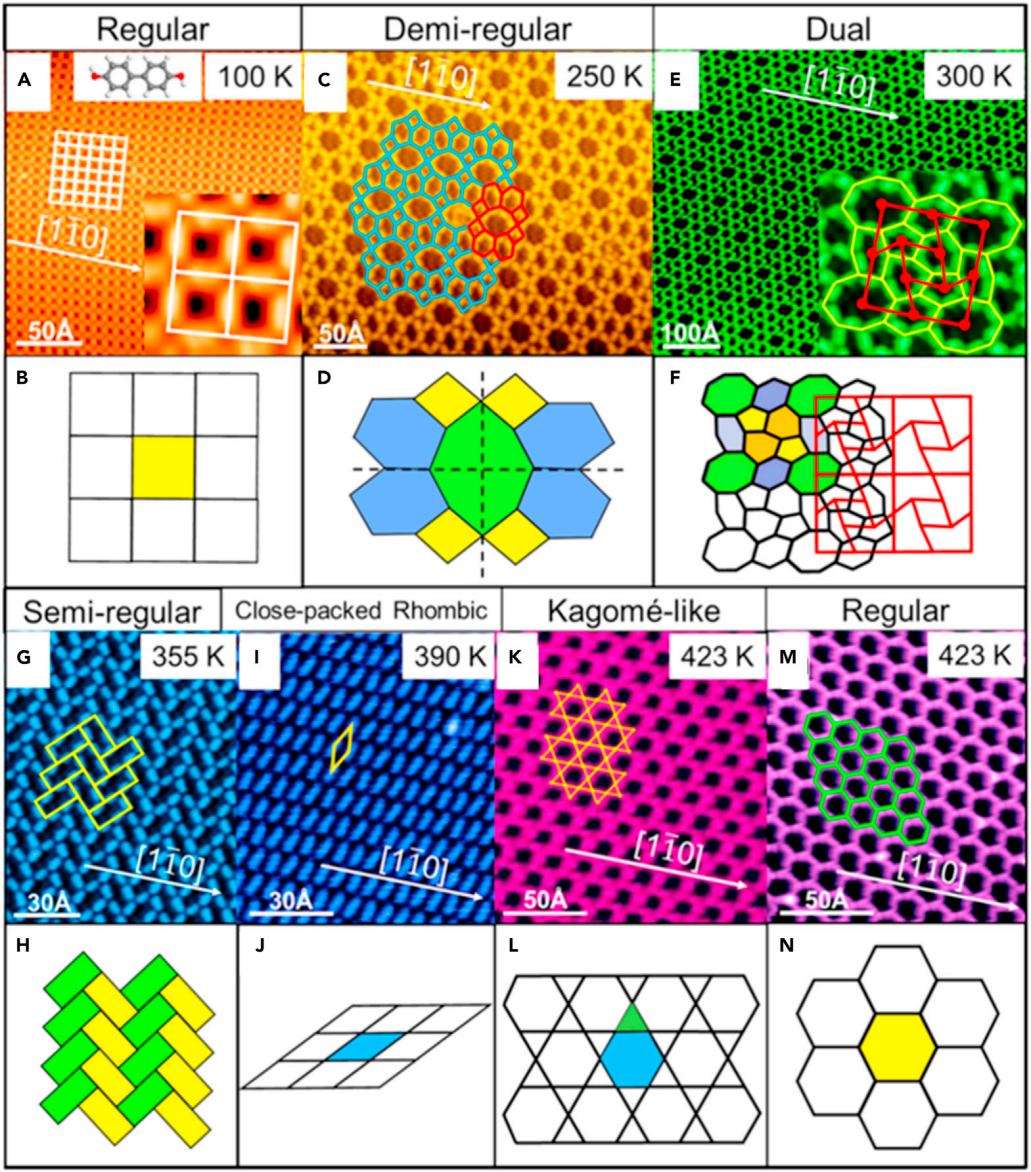
Supramolecular structure of 4,4′-dihydroxybiphenyl molecule on Ag(111) substrate and STM diagram after annealing at different temperatures STM images obtained (A) after deposition of DHBP on Ag(111) held at 100 K, followed by the stepwise annealing at (C) 250 K, (E) 300 K, (G) 355 K, (I) 390 K, and (K abd M) 423 K. Corresponding schematic diagram of each structure are displayed below each STM image (B, D, F, H, J, L, and N). Figures reproduced from: Feng et al.^79^ Copyright 2019 American Chemical Society.

The symmetrical molecule with endmost −OH at each end is a perfect building structural unit for exploring the effect of molecular configuration on the symmetry of coordination compound. With each H atom removed, the hydrogen bonds between molecules must be realigned. This resulted in an interesting “desymmetrization−symmetrization” dynamic transformation of acme symmetry in the coordination compound. Zhu et al. deposited 4,4′-dihydroxybiphenyl on the Ag(111) substrate held at 100 K and then annealed to attain the progressive dehydrogenation of the endmost −OH.^79^ Figure 7 showed the STM pattern of molecules deposited at 100 K and annealed at 299, 354, 389 and 422 K, respectively. The surface molecular chemical structure and the dynamic transformation of self-assembled structure on the substrate were studied by STM and SRPES. Several regular and orderly assembly structures were formed during the annealing process. In these unprompted formed patterns, the dynamic transformation, then later rebuild of the vertex of an excellent symmetry, was noticed with increasing the temperature of metallic substrate. This work inspired a new wave of studies of building molecular vertices on metal substrates, and increased the complexity of architecture for the development of functional surfaces.

Designing new reaction types and corresponding reaction paths are effective ways to achieve structural and functional diversity. Although on-surface chemistry has made great progress in the past few years, it is still challenging to design new reaction types. Carboxyl groups can form complex bonds with metal surface atoms to increase their adsorption capacity and selectivity. It can also introduce negative charge on the metal surface to enhance its electrochemical activity and corrosion resistance. In addition, carboxyl groups can undergo nucleophilic substitution reactions with different nucleophiles, thus allowing the metal surface to further functionalize with various molecules. Gao et al. used decarboxylative coupling of aromatic diacids to obtain polymeric bisnaphthyl-Cu species at high temperature as intermediate products.^80^ Compared with the metal surface of other crystallographic plane, Cu(111) surface worked most efficiently to guide such the polymerization of molecules. The further increase of reaction temperature led to the establishment of polymer through decarboxylation, which was reduced and eliminated after further annealing to ultimately generate poly-naphthalenes.

The deposition of 4-Azidobenzoic acid (ABA) on the surface of Ag and Cu single crystal substrates will lead to the decomposition of azide groups, and the release of a N_2_ molecule per ABA molecule.^81^ Different physical properties and crystal orientation of the metal surface will cause these coordination dimers to form SMOFs with different coordination modes. On Ag(111) surface a complex structure were constructed with relatively strong coordination. On Ag(100) surface, 2-fold symmetry coordination chain structure were formed, and after annealing, and then transformed into a fully coordinated network structure with quadruple symmetry after annealing. On Cu(100) surface, four Cu atoms could be captured as coordination centers to form new coordination complexes. This study expanded the types of traditional on-surface chemistry reaction and enriches the diversity of SMOFs structures.

**Figure 8:**
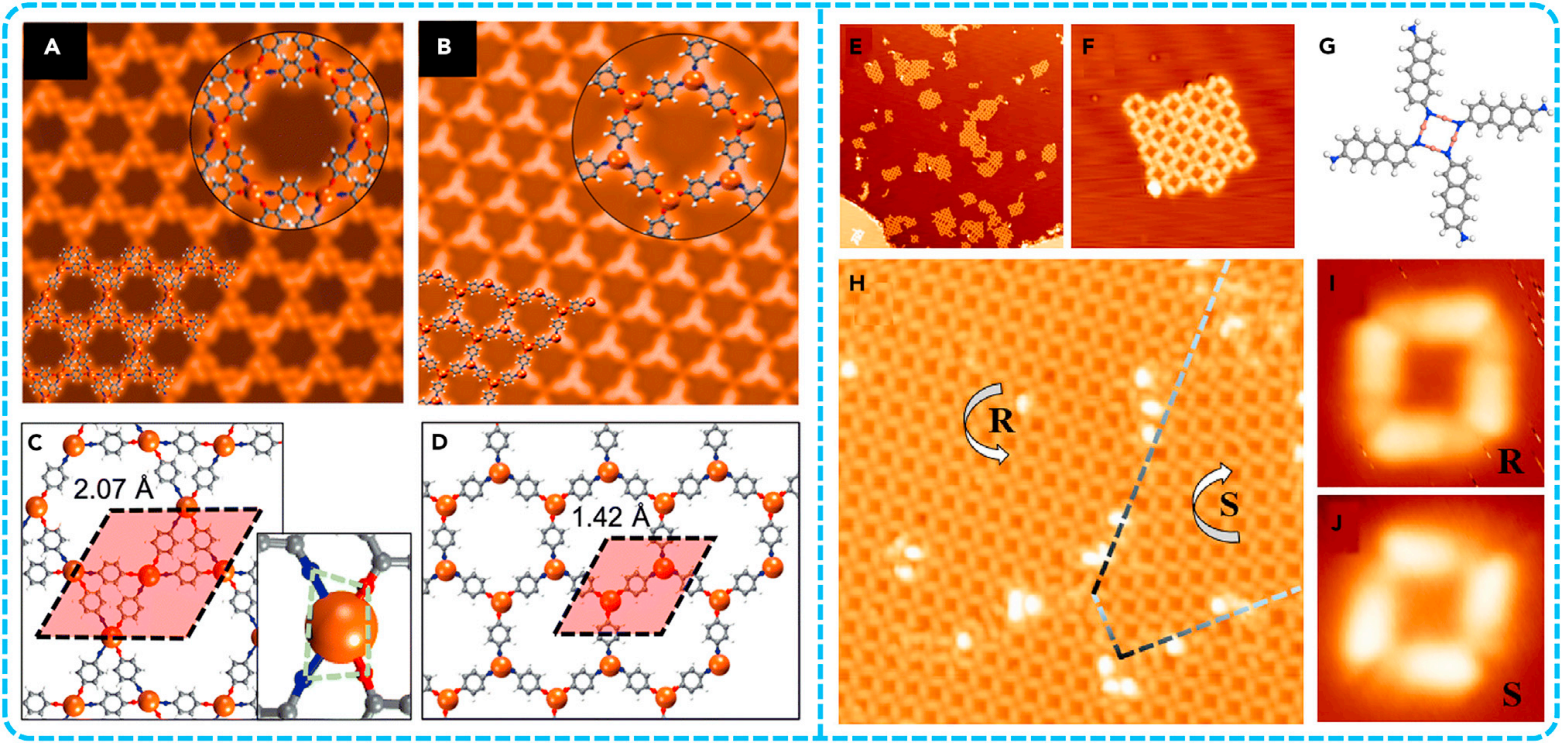
Computed Keldish-Green STM images (A) Kagome and (B) Honeycomb structures at constant-current regime. Pictorial top view of the optimized Kagome (C) and honeycomb (D) network structures. Figures reproduced from: Ruiz et al.^82^ Copyright 2023 Royal Society of Chemistry. (E) STM image of molecules deposition onto Cu(100) surface held at RT, followed by annealing at 370 K. (F) STM image of an isolated domain. (G) Structural model of the porous network. (H) STM image composed of two domains. (I and J) Two individual square tetramers with different chirality. Figures reproduced from: Song et al.^83^ Copyright 2023 American Chemical Society.

During the construction of SMOFs, the final structure is governed by a competition between dynamical and thermodynamic mechanisms. Different experimental parameters, such as surface temperature, deposition sequence, annealing temperature or surface atomic density, are important factors in determining reaction efficiency. For example, it is evident that surface diffusion is hindered by annealing a highly covered surface or by employing low temperatures during deposition, and the resulting molecular structure can be stabilized as a metastable phase. Furthermore, different coverages of precursor on the surface will affect other factors such as the collision frequency, rotation degree and surface diffusion of the molecular activated species, and further directly determine the reaction process. The network structure formed by *para*-aminophenol molecules on Cu(111) surface could be adjusted to Kagome or honeycomb lattice through a kinetic or thermodynamically controlled reaction (Figures 8A and 8B).^82^ The optimized network structure revealed that coordination bonds formed between N/O atoms and Cu atoms dominated the establishment of SMOF (Figures 8C and 8D). The oxidation process of precursor molecules on the surface was completely dependent on the final temperature (thermodynamics). The formed SMOF structure depended on the particular experimental sequence (kinetics). By fine-tuning the molecular coverage and heat treatment temperature, the hierarchical dehydrogenation of anthracene-2,6-diamine (ADA) precursor molecules on Cu(100) surface were successfully achieved, showing large scale SMOF with low defect density.^83^ The separation of the first H atom of the amino group resulted in the formation of a separated tetramer oligomer via the N−Cu−N bond (Figure 8E−8G). The tetramers were interconnected to form SMOF after annealing at 370 K (Figure 8H). Furthermore, the high-resolution STM revealed two independent square tetramer structures with different chirality (Figures 8I and 8J). These results emphasized the importance of the balance between thermodynamics and dynamics in the final structure of SMOF.

## Geometric topological structure of SMOFs

SMOFs are 2D layer lattices formed by the plane coordination between metal nodes and organic ligands. In the past few decades, SMOFs with different lattice structures, including honeycomb structure, Kagome lattice, hexagonal geometry and Sierpiński triangles, have been fabricated from different combinations of metal atoms and organic molecules.

### Honeycomb structure

**Figure 9:**
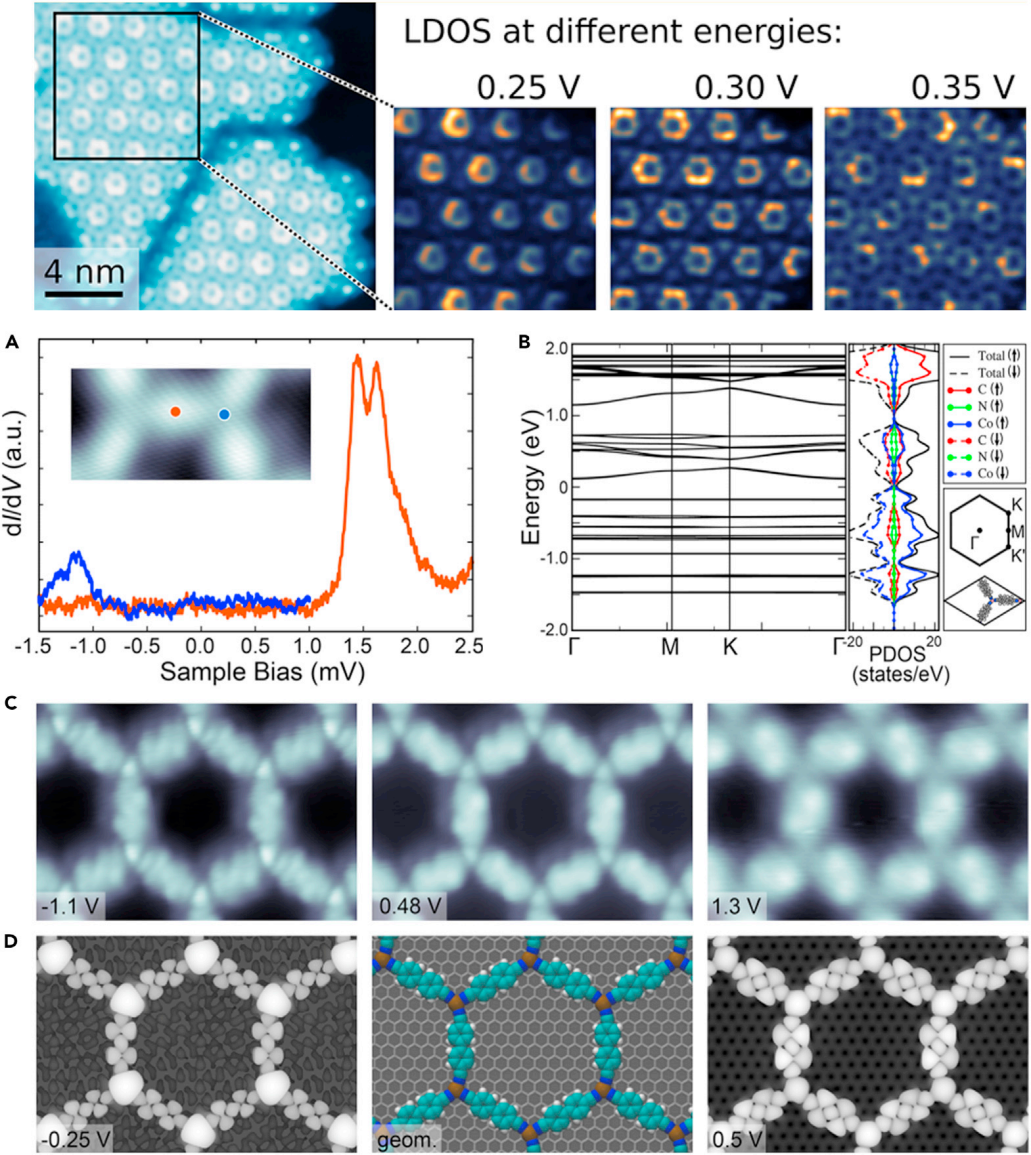
STM images and electronic properties of honeycomb SMOF (A) Scanning tunneling spectroscopy at the marked position shown in the image. (B) Calculated band structure and gross PDOS of the 2D assembly structure. STM for (C) experimental and (D) simulation calculation respectively, scan size is 6.2 × 4 nm^2^. Figures reproduced from: Kumar et al.^84^ Copyright 2018 American Chemical Society.

Different organic ligands (dicyanobiphenyl, 4,4′-dicyanobiphenyl, DCBP, dicyanoanthracene, DCBP, and 9,10-dicyanoanthracene, DCA) with cobalt atoms could control the establishment of stable SMOFs on epitaxial graphene (Figure 9).^84^ The inherent electronic properties of SMOFs were obtained based on STM, AFM, STS and DFT calculations. There was only weak coupling between the building units of DCBP-MOF, and DCA-MOF showed significant in-plane hybridization, forming a 2D electronic state with large bandwidth (Figures 9A and 9B). The occupied states had more remarkable metal character contrasted to the unoccupied states, which were mostly consisted of the ligand states (Figures 9C and 9D). This work opened the experimental path toward the design and synthesis of SMOFs-based electronic materials with complex and engineering structures.

Understanding the relationship between coordination configuration and electron state at the microsystem is crucial. Linear biphenyl molecule and Fe atoms were assembled on Au(111) substrates to form a honeycomb SMOF (Fe-DPBP).^15^ There were multiple coordination modes Fe atoms in the metal−organic coordination network. Interestingly, as the coordination number increases, the hybrid state transfers to a higher energy, which opens up experimental routes to explore the interaction between organic ligands and metal atoms.

Isocyanides are crucial organic building molecule and has attracted much attention in recent years. It can be used to make polyurethane products, such as foams, coatings, adhesives and sealants, which have high strength, durability and flexibility. Different from other coordination elements, the terminal C atom of isocyanate contains special lone pair electrons and empty orbitals, which is beneficial to combine with metal substrate atoms to generate C−M bond. Therefore, the excellent binding ability of isocyanates on metal substrates provides a prerequisite for the construction of SMOFs with unique geometry and function. In 2021, Liu et al.^85^ obtained a honeycomb SMOFs consisting of 3-fold (isocyano)3-Cu motifs by coordination and self-assembly of 1,4-phenylene diisocyanobenzene on Cu(111) surface. The structure of SMOFs was controlled by surface stereochemistry, including the adsorption conformation of isocyanide molecules and the arrangement of the action sites. Nevertheless, it was difficult for *m*-DICB to form MOF because of its fixed conformation on Cu(111) metal substrate.

Besides, halogen atoms have a significant effect on the formation of metal−organic coordination motifs on the surface. Embedding halogen atoms into metal−organic coordination systems has far-reaching guiding significance for enriching surface structure diversity. Xu et al.^86^ explored the universality of halogen atoms used to regulate SMOFs employing 9-ethylguanine molecule and Ni atoms as the building unit. Iodine atoms were uniformly located in specific hydrogen-rich locations surrounded by 9-ethylguanine through electrostatic interaction, which was the key to structural stability. Importantly, the strategy was universal in halogen solutions of multiple systems. These findings disclosed new avenues to the further exploration of the promotion of halogens as functional building elements.

### Kagome lattice

Kagome lattice is a conjugated lattice of honeycomb structure, which is a special structure composed of opposite triangular lattice. Kagome lattice retains the Dirac band of honeycomb structure, which makes Kagome lattice a good platform for studying flat band and Dirac band. In addition, spin frustration may occur when the atoms of the triangular lattice in the Kagome lattice are magnetic. The increasing development of on-surface synthesis provided new opportunities toward the fabrication of Kagome lattices, which were usually stabilized by intermolecular interactions.

**Figure 10:**
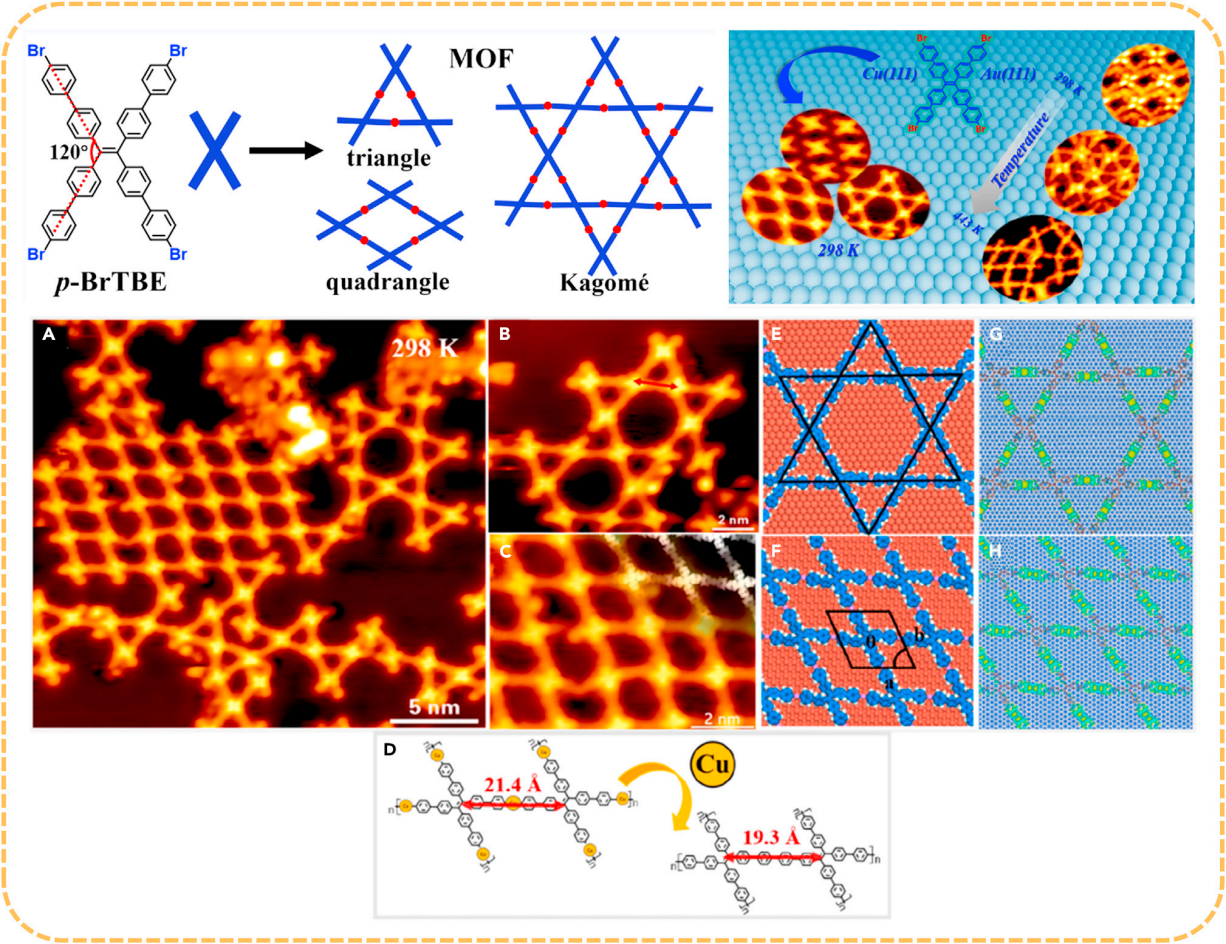
Construction of SMOF with Kagome lattice (A) Large-scale STM image showing the metal−organic network. (B) High-resolution STM image for the Kagomé structure. (C) The ordered quadrangle pattern observed by STM. (D) The process of transition from organometallic intermediates to covalent bonds. Theoretical simulation diagram: (E) Kagomé structure, (F) parallelogram structure. The charge difference diagram: (G) Kagome structure, (H) quadrilateral structure. Figures reproduced from: Miao et al.^87^ Copyright 2023 American Chemical Society.

Compared with non-covalent interactions such as van der Waals force, hydrogen bonding and π-π stacking, metal-organic coordination bonds have the advantages of ideal selectivity, high directivity and self-healing ability due to the reversibility. In addition, metal−organic coordination bonds can not only act as a bridge for intermolecular charge transfer, but also improve the thermal stability of the Kagome lattice. In recent years, the Kagome lattice of SMOFs based on different systems have been fabricated. In 2023, Miao et al.^87^ introduced *p*-bromine units into tetraphenylethylene molecule in order to construct the SMOF with the Kagome lattice on Au(111) and Cu(111) surfaces. The significant discrepancies in self-assembling nanostructures between Au and Cu surfaces were illustrated by means of STM and DFT calculations. The catalytic properties and lattice arrangement of metals are the key reasons for the formation of structural diversity (Figure 10). This work provided a new mechanism for the formation of SMOFs at the atomic scale, and thus conducive to the construction of high-precision electronic devices and new nanomaterials.

SMOFs with Kagome lattice exhibits rich magnetic behaviors, such as spin glass and spin liquid. Kagome antiferromagnetic (KAF) lattice is a kind of magnetic material with special lattice structure, which has attracted many researchers’ interest. Importantly, KAF has novel and unique physical and chemical phenomena, which indicates that it is a potential spintronic material. In 2021, Lin et al. designed and synthesized an SMOF layer and resolved the atomic structure of the Kagome Fe(II) lattice.^88^ The theoretical simulation results show that Fe^2+^ were in a higher spin state. In addition, spin excitation at 6 meV was observed using tunneling spectra. Replacing Cu (II) with Fe (II) of the same structure opened the experimental path toward realizing spin 1/2 KAF.

The electronic materials with Kagome lattices exhibit special energy band structures, which are expected to be used to adjust the topology and construct strong correlation electronic phases. A Kagome lattice SMOF, Fe_3_(HITP)_2_, was designed and synthesized on Au(111) substrate, which comprised of a Kagome sublattice of Fe atoms.^89^ The structure of SMOF was analyzed by STM and STS at single molecular resolution, and it was found that SMOF retained the electronic and magnetic properties of independent framework. DFT results showed that the structure exhibited a ferromagnetic ground state with extraordinary band gap. Subsequently, a convincing experimental result for the magnetic moment effect caused by the electron coulomb interaction in SMOF was reported.^90^ The Kagome structure was composed of di-cyano-anthracene (DCA) molecules and Cu atom on Ag(111) surface. Measurements of the Kondo effect revealed that the electrons in the Kagome lattice were strongly coulombically influenced by each other, resulting in magnetism. Nanoelectronics and spintronics techniques based on adjustable related electronic phases in SMOFs were enabled by this result.

**Figure 11:**
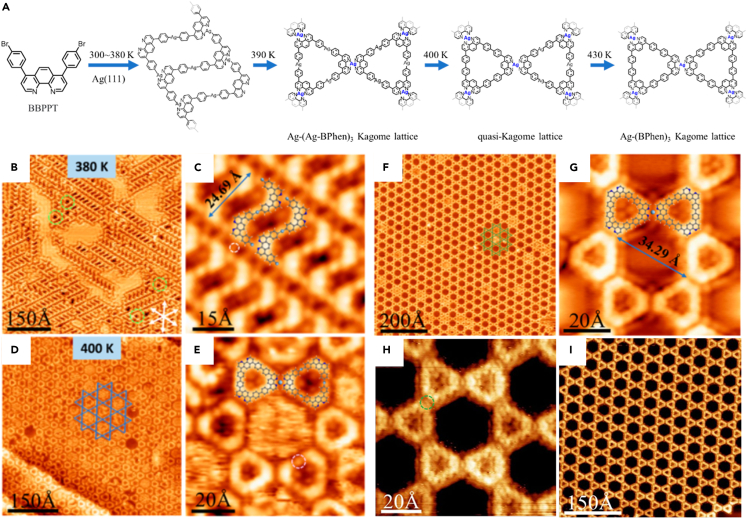
Construction of a large area and defect-free Kagome lattice SMOF (A) Reaction process of the construction of N−Ag coordinated Kagome nanostructures on Ag(111). (B) Large-scale STM image of the OM coordination ladder structures after annealing the sample to 380 K. (C) High-resolution STM image of ladder structures. (D) Large-scale STM image of quasi-Kagome lattice after annealing at 400 K. (E) High-resolution STM image of trimer macrocycle. (F) Large-scale STM image of the covalent Kagome lattice. (G) High-resolution image of panel *a* overlaid. (H) High-resolution STM image of Kagome lattice scanned using a sharp tip. (I) Large-scale STM image of Kagome lattice after annealing at 520 K. Figures reproduced from: Xiong et al.^91^ Copyright 2023 American Chemical Society.

Currently, it is still a challenge to construct large area and defect-free Kagome lattice SMOFs. A inspiring strategy for on-surface synthesis was employed to construct two large-scale SMOFs consisting of 4-fold N−Ag bonds on the Ag(111) substrate.^91^ For example, the quasi-Kagome lattice with two different size nodes and the Kagome lattice was formed by covalent trimers (Figure 11A). The excellent selective construction of Kagome lattice was a thermodynamically controlled reaction process. Due to the reversible C−Ag−C bonds, the ladder structure tended to transform into a thermodynamically stable trimer macrocycle (Figures 11B−11E), which immediately assembled into a stable quasi-Kagome structure (Figures 11F−11I). This strategy opened up a feasible way to fabricate new 2D supramolecular nanostructures on the surface, which may show unique physical and chemical properties.

### Hexagonal geometry

A large number of hexagonal MOF networks are constructed on the surface by selecting suitable metal clusters and organic ligands. Dou et al.^92^ designed layered MOFs with hexagonal grids. It was stabilized by triangular molecules and square plane mononuclear metal sites, which made the electronic structure of SMOF achieve excellent electron delocalization through continuous conjugation, showing remarkable metal properties.

Wang et al.^93^ constructed a self-assembled 2D chiral network over an extensive area of Au(111) and Ag(111) after deposition of the prochiral precursor, 6,12-dibromochrysene (DBCh). In the Ullman coupling process, the DBCh enantiomers were induced into hexagonal channels with self-assembly on both Au(111) and Ag(111) surfaces, and organometallic (OM) oligomers with local chiral properties were formed on Au(111) and Ag(111). This result not only provided solid evidence for the precise preparation of SMOFs via a feasible bottom-up method, but also disclosed new avenues for the comprehensive study of chirality.

**Figure 12:**
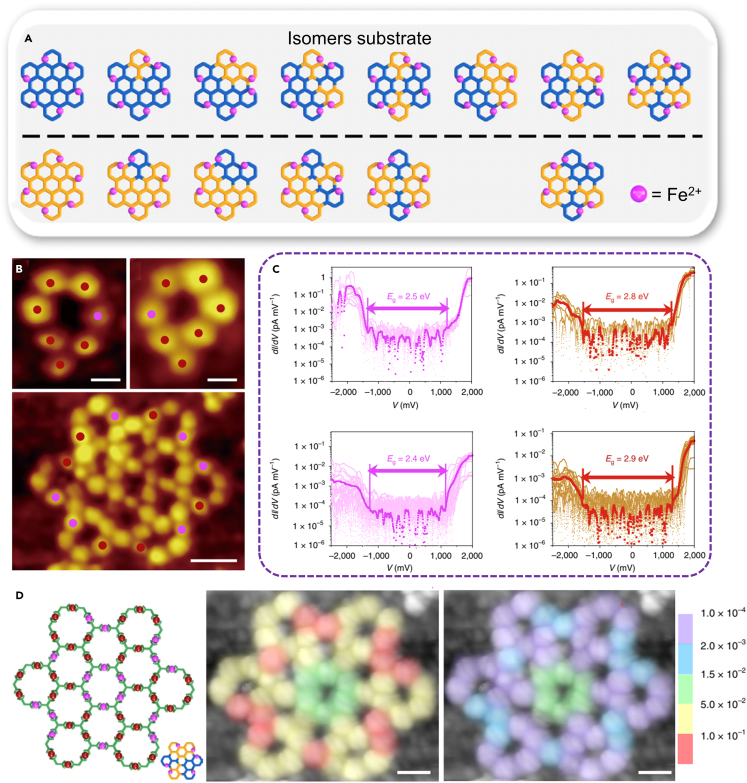
Construction of SMOF with hexagonal grid (A) A variety of possible isomers that may exist on Ag(111) substrate. (B) STM images of two kinds of SMOFs with different structures. (C) Local conductance voltage tunneling spectrum of Fe^2+^. (D) A model of the hexagonal grid. Figures reproduced from: Zhang et al.^94^ Copyright 2020 Nature Publishing Group.

In order to realize the assembly of nanostructures with large area and clear configuration, Zhang et al.^94^ combined intermolecular and intramolecular forces to successfully construct a metal−organic grid with a width of more than 20 nm, and possess eight isomers based on different substructure orientations. (Figures 12A and 12B). The reaction intermediates and the resulting metal−organic grids were investigated by STM and STS at submolecular resolution (Figures 12C and 12D). This work was of great value for the characterization and identification of molecular isomers at the atomic level.

The design of 2D metal−organic network via selective lanthanide metal provides a wise and effective strategy to construct a wide range of SMOFs architectures such as porous frameworks with expected topological geometry and excellent structural stability. Lin et al. reported the self-assembly of lanthanide metal Eu with two heterotypic ligands on the Au(111) surface.^95^ The Eu central atoms were connected in 4-fold or 5-fold coordination schemes by terpyridyl and two carbonitrile ligands to form 1D sinusoidal chains or 2D molecular porous networks. Specific ligand stoichiometry could achieve more than 95% self-assembly pattern selectivity. This indicated that surface−confined lanthanide metals provide a common and flexible coordination motif for the design of SMOFs.

### Sierpiński triangles

A fractal is “a rough or fragmented geometric shape that can be split into parts, each of which is a reduced-size copy of the whole” as proposed by Benoit B. Mandelbrot in 1982.^96^ Fractals show similar properties on each scale, and it has profound significance in geometric topology, mathematics, art and other fields, and is widely found in nature and in real life, such as snowflakes, lightning and coastlines.^97^ The generation of molecular fractals is mainly concentrated in synthetic chemistry, and the construction of molecular fractals through surface molecular self-assembly strategies remains challenging. In order to investigate the formation process of these complex and fascinating constructions, researchers have made great efforts to design and fabricate molecular fractals in previous decades. Sierpiński triangle (ST) is a representative fractal structure, which has the overall shape of an equilateral triangle and is internally subdivided into smaller equilateral triangles.^98^ Interestingly, any part of it has the same structure as the whole and is expected to exhibit distinctive optical and electromagnetic properties.^99^

To construct the larger size SMOFs with ST structure, 120° V-shaped molecules and 3-fold nodes are essential. Increasing the bonding strength of the sites can overcome the limitation of surface symmetry, resulting in STs formation on non-triple substrates. The cyano (−CN) group and Fe can form 3-fold bonding on single-crystal surfaces. Li et al.^100^ employed 4,4″-dicyano-1,1’:3′,1″-terphenyl molecules and Fe atoms to construct Sierpiński triangles on Au(111) surfaces. The whole system of defect-free fractal crystals pertains to the C_3v_ point group. The structure was analyzed by low-temperature STM and DFT calculation. The establishment of surface-supported fractal crystals was revealed via Monte Carlo simulation, which was attributed to the 3-fold nodes dominating the establishment of fractal crystal structure.

**Figure 13:**
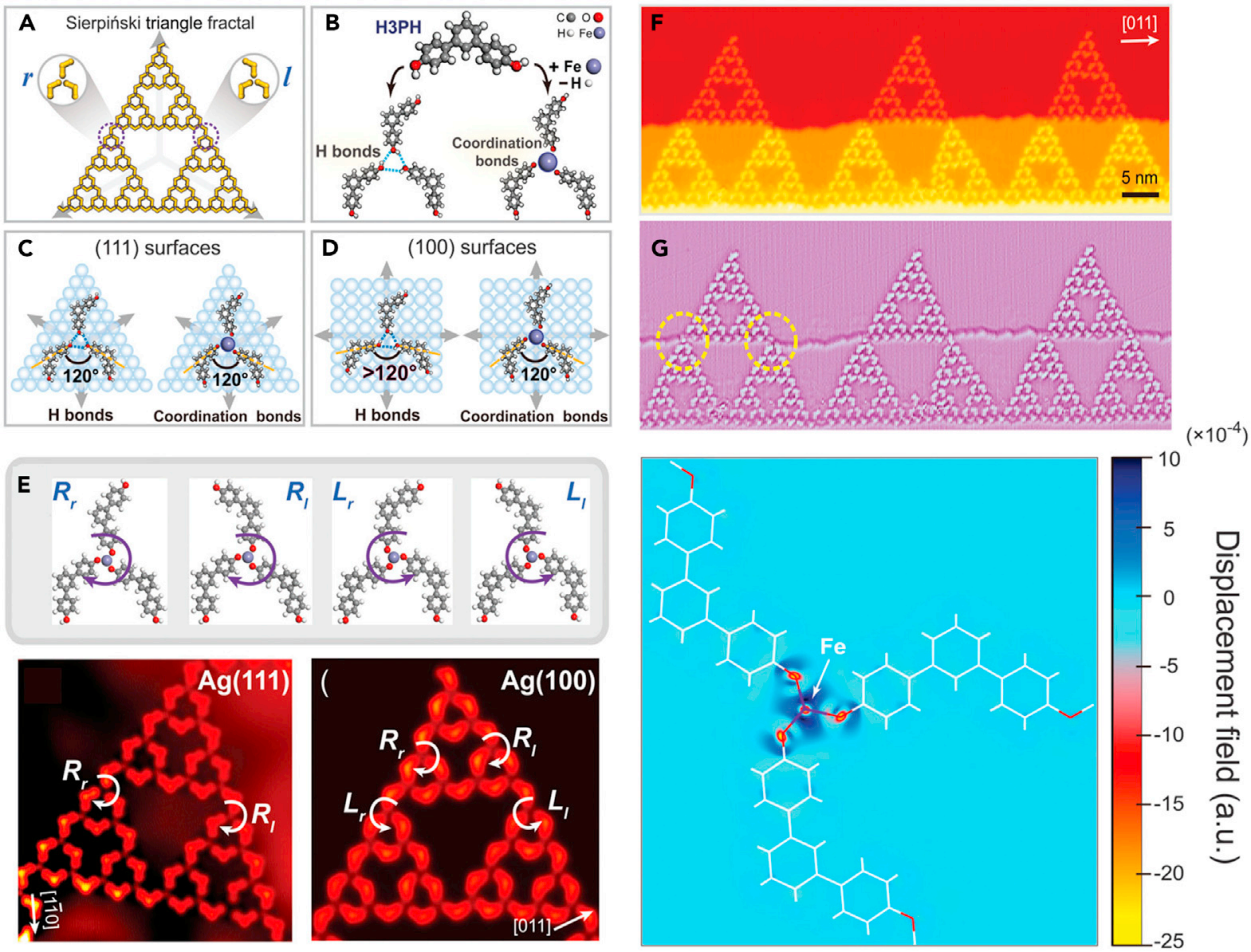
Construction of defect-free ST fractal structure (A) Schematic diagram of the construction module of ST fractal. (B) Construction of ST structure on Ag substrate based on hydrogen bond and coordination Bond. The trimer model formed on the (C) Ag(111) and (D) Ag(100) surface. (E) Several chiral structures in Fe−O coordination system. STM image of ST fractal crossing a step on Ag(100) (F) before and (G) after the Laplace transformation. Figures reproduced from: Zhang et al.^101^ Copyright 2016 Royal Society of Chemistry.

Zhang et al.^101^ constructed a defect-free ST fractal on both Ag(111) and symmetry-mismatched 4-fold Ag(100) substrates using 4,4″-dihydroxy-1,1’:3′,1″-terphenyl (H_3_PH) molecules and Fe atom under vacuum. The fractal order was up to 3 (Figure 13A). The trimer used to construct ST fractals could be connected by hydrogen bonds or coordination bonds (Figure 13B). Figure 13C was the model of hydrogen bond and coordination bond trimer on 3-fold (111) surface. The trimer structure formed by the combination of two forces on a 4-fold (100) surface was shown in Figure 13D. The DFT calculation demonstrated that the strong coordination interaction between Fe and O atoms stabilized the fractal structure (Figures 13E−13G). Moderately strong hydrogen bonds allowed pure H_3_PH molecules to form STs only on Ag(111), not on Ag(100), as opposed to other cases.

**Figure 14:**
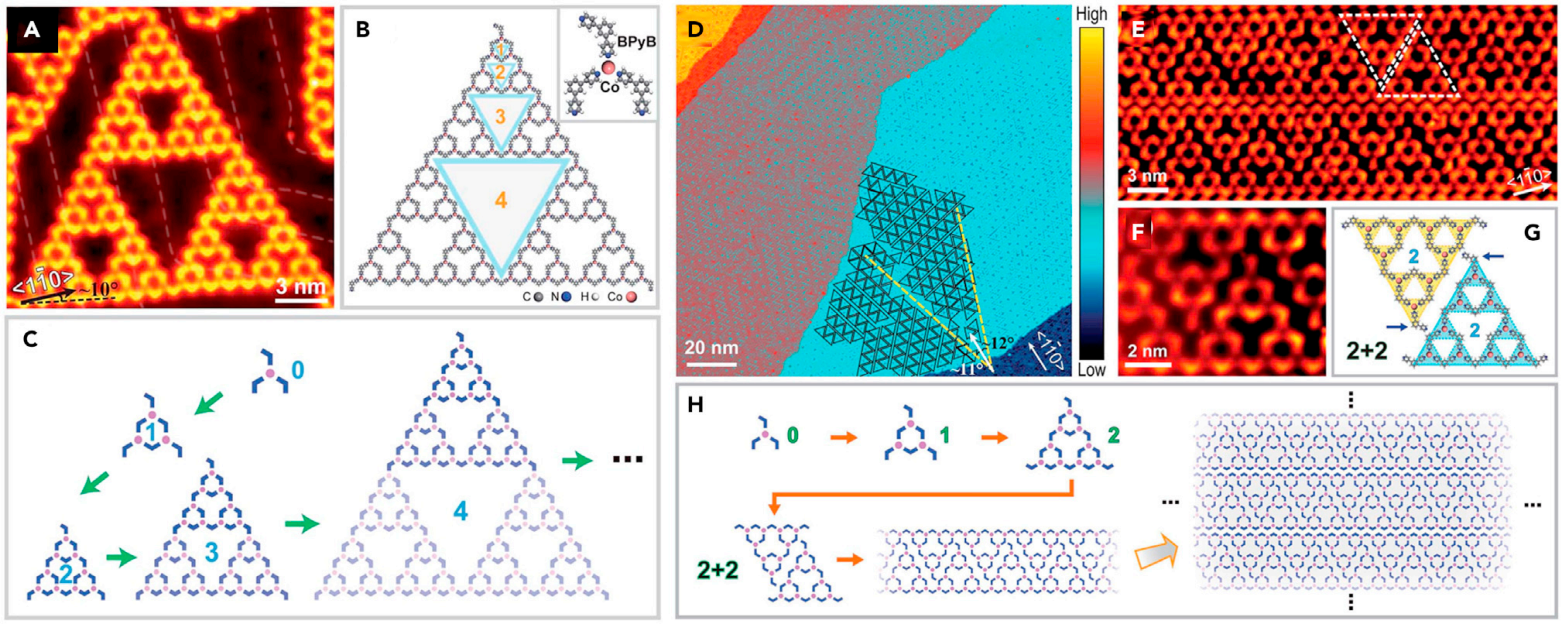
Construction of defect-free ST fractal structure (A) STM image of fourth-order defect-free ST. (B) Model diagram of the ST fractal. (C) A schematic diagram of the process of gradually forming fractals from construction primitives. (D) Molecular ribbons constituted by close-packed order fractal structure. (E) Image of closely packed molecular bands. (F) The STM image of repetitive unit. (G) The corresponding model structure of repetitive unit. (H) Schematic diagram of the formation process of large area network structure based on ST fractal. Figures reproduced from: Zhang et al.^102^ Copyright 2018 Royal Society of Chemistry.

Subsequently, they constructed a SMOFs of STs structure on Au(111) surface by 120° V-shaped 1,3-bi(4-pyridyl) benzene (BPyB) and Co atoms, and studied by low-temperature STM.^102^ The STM pattern with a defect-free ST of 4 order and the structure mode of ST were shown in Figures 14A−14C. At a low coverage, higher-order STs were discovered. At a high coverage, the STs displaying different types of 1D chains were formed (Figures 14D−14F). The generation of long chain structure resulted in the higher coverage and matching (Figure 14H).

In recent years, STs was found to possess special optical and electronic properties. To investigate their properties and reveal practical application value, Wang and co-workers used molecular design and epitaxy control to pack ST into large-scale 2D crystal structures.^103^ Various ordered structures consist of ST units were successfully synthesized on the Au(111) surface via the coordination of 1,3-bis(4-pyridyl) benzene molecules and Fe atoms. When the coverage of organic molecules was low, the fifth-order BPyB-Fe-ST pattern was clearly observed. With the increase of coverage, the interesting arrangements of ST- (2–2), ST- (1–2), ST- (1–1) and ST- (0–2) were established. The size and symmetry of STs matched exactly with the triple Au(111) surface lattice, which eliminated the structural error and improved the stability of large-scale STs crystal. Subsequently, DFT calculation and K-map were utilized to quantitatively analyze the effect of surface epitaxy on the formation of STs.

## Conclusions and outlooks

The survey of results presented in this review exemplifies the abundant potentialities of SMOFs for directing topological diversity, enriching surface reaction types, and realizing functional applications. STM and quantum technologies have laid a solid foundation for the field to flourish. The regulation of structural diversity of SMOFs induced by extrinsic transition metal, alkali metal and lanthanide metal atoms are first introduced. Based on the special arrangement of *d* and *f*-blocks of electrons, atomic size and spin-orbit coupling reveal different physical and chemical properties. Simultaneously, the substrate−assisted SMOFs were discussed in depth around Ullmann coupling, the dehydrogenation of carboxyl, hydroxyl, and sulfhydryl groups, and the decomposition of azide groups. Subsequently, the complex and fascinating periodic and fractal structures formed by on-surface self-assembly are summarized, including honeycomb structure, Kagome lattice, hexagonal geometry and Sierpiński triangles, and determine the related prospects of designing functional nanoscale systems and construction. These findings herald multiple promises for SMOFs in design and synthesis, structure and function regulation, and even practical application in the future.

In recent years, although SMOFs has made outstanding research achievements and breakthroughs, and obtained fascinating topological geometry and physical and chemical properties, it still faces many challenges in the future development process. At present, most of the construction of SMOFs is Ullmann coupling reaction. It is urgent to design a new type of surface reaction, which is the key to further expand the structure and function of SMOFs. On the other hand, the pathway of the surface−confined coupling reaction, the mechanism of surface activation and the change of the charge state of metal atoms in the reaction are largely unknown. The relationship between the molecular structure of precursor molecules and the crystal packing of intermediate and final products cannot be predicted *a priori* due to the current limitations of 2D crystal engineering. In addition, many central questions about reactivity, chemical selectivity and regional selectivity of certain surface reactions are still poorly understood, which hinders more precise regulation in on-surface synthesis that is essential for enhancing the structural stability of the insufficient, and is the most critical obstacle for current applications.

By overcoming these enticing challenges and utilizing the precious opportunities presented in the field, the significant developments for multitudinous disciplines and sciences in the 21st century seem assured, providing plentiful prospects for the development of the basic disciplines and the application of science and technology.
